# Genome-wide association study of periodontal pocketing in Finnish adults

**DOI:** 10.1186/s12903-021-01964-8

**Published:** 2021-11-30

**Authors:** Paula Tegelberg, Jussi Miikkael Leppilahti, Atte Ylöstalo, Tellervo Tervonen, Johannes Kettunen, Anna Liisa Suominen, Pekka Ylöstalo

**Affiliations:** 1 Research Unit of Oral Health Sciences, Faculty of Medicine, University of Oulu, Oulu, Finland; 2 Computational Medicine, Faculty of Medicine, University of Oulu, Oulu, Finland; 3 Center for Life Course Health Research, Faculty of Medicine, University of Oulu, Oulu, Finland; 4 Biocenter Oulu, University of Oulu, Oulu, Finland; 5 Medical Research Center, Oulu University Hospital and University of Oulu, Oulu, Finland; 6 National Institute for Health and Welfare, Helsinki, Finland; 7 Department of Oral and Maxillofacial Surgery, Oulu University Hospital, Oulu, Finland; 8 Institute of Dentistry, University of Eastern Finland, Kuopio, Finland; 9 Department of Oral and Maxillofacial Surgery, Kuopio University Hospital, Kuopio, Finland; 10 Department of Public Health and Welfare, National Institute for Health and Welfare, Helsinki, Finland

**Keywords:** Epidemiologic studies, Genome-wide association study, Periodontal pocket, Periodontitis

## Abstract

**Background:**

A genome‐wide association study is an analytical approach that investigates whether genetic variants across the whole genome contribute to disease progression. The aim of this study was to investigate genome-wide associations of periodontal condition measured as deepened periodontal pockets (≥ 4 mm) in Finnish adults.

**Methods:**

This study was based on the data of the national Health 2000 Survey (BRIF8901) in Finland and the Northern Finland Birth Cohort 1966 Study totalling 3,245 individuals. The genotype data were analyzed using the SNPTEST v.2.4.1. The number of teeth with deepened periodontal pockets (≥ 4 mm deep) was employed as a continuous response variable in additive regression analyses performed separately for the two studies and the results were combined in a meta-analysis applying a fixed effects model.

**Results:**

Genome-wide significant associations with the number of teeth with ≥ 4 mm deep pockets were not found at the p-level of < 5 × 10^−8^, while in total 17 loci reached the p-level of 5 × 10^−6^. Of the top hits, SNP rs4444613 in chromosome 20 showed the strongest association (*p* = 1.35 × 10^−7^).

**Conclusion:**

No statistically significant genome-wide associations with deepened periodontal pockets were found in this study.

## Background

According to the current conception, susceptibility to the initiation and progression of periodontitis depends on the strength of the genetically determined immune response against bacteria [1, 2]. Modifying co-factors associated with periodontitis include age, sex, smoking, diabetes, obesity, and socioeconomic factors [3, 4].

Using a classic twin study design, Michalowicz and his group [5, 6] showed that approximately 50% of the variation in clinically determined adult periodontitis was attributed to genetic variance. A later twin study, which used self-reported data on periodontal condition, reported lower heritability estimates for periodontitis; 39% in women and 33% in men [7]. Overall, heritability seems to be higher for severe early-onset traits and among younger individuals [2, 8].

Although the twin studies were crucial in determining the overall role of the genetic component behind periodontitis they could not be used for the identification of the number and location of the susceptibility genes. To achieve the latter goal, two widely used approaches have been used; a candidate gene association study and a genome-wide association study (GWAS). In a recent systematic review and meta-analysis, the authors concluded that up to one-third of periodontitis variance in the population was attributable to genetic factors [2]. In general, lower heritability was reported in studies using GWAS design in comparison to twin studies or other family studies [2].

GWAS enables screening of genetic variants across the whole genome linked to a disease under investigation. Unlike the candidate gene studies, in which the selection of target genes is based on their known role in the pathogenesis of the disease, GWAS offers an approach for the determination of novel polymorphisms that is not limited by prior knowledge. A number of studies have investigated genome-wide associations of different periodontitis phenotypes [2, 9, 10]. In terms of the GWA approach in chronic periodontitis, several studies using various case definitions and populations of different ethnic origins have been published [8, 11–22]. Only a few of these studies reported associations on the level of stringent genome-wide significance level (*p* value < 5 × 10^−8^) [18, 20–22].

## Methods

**Fig. 1:**
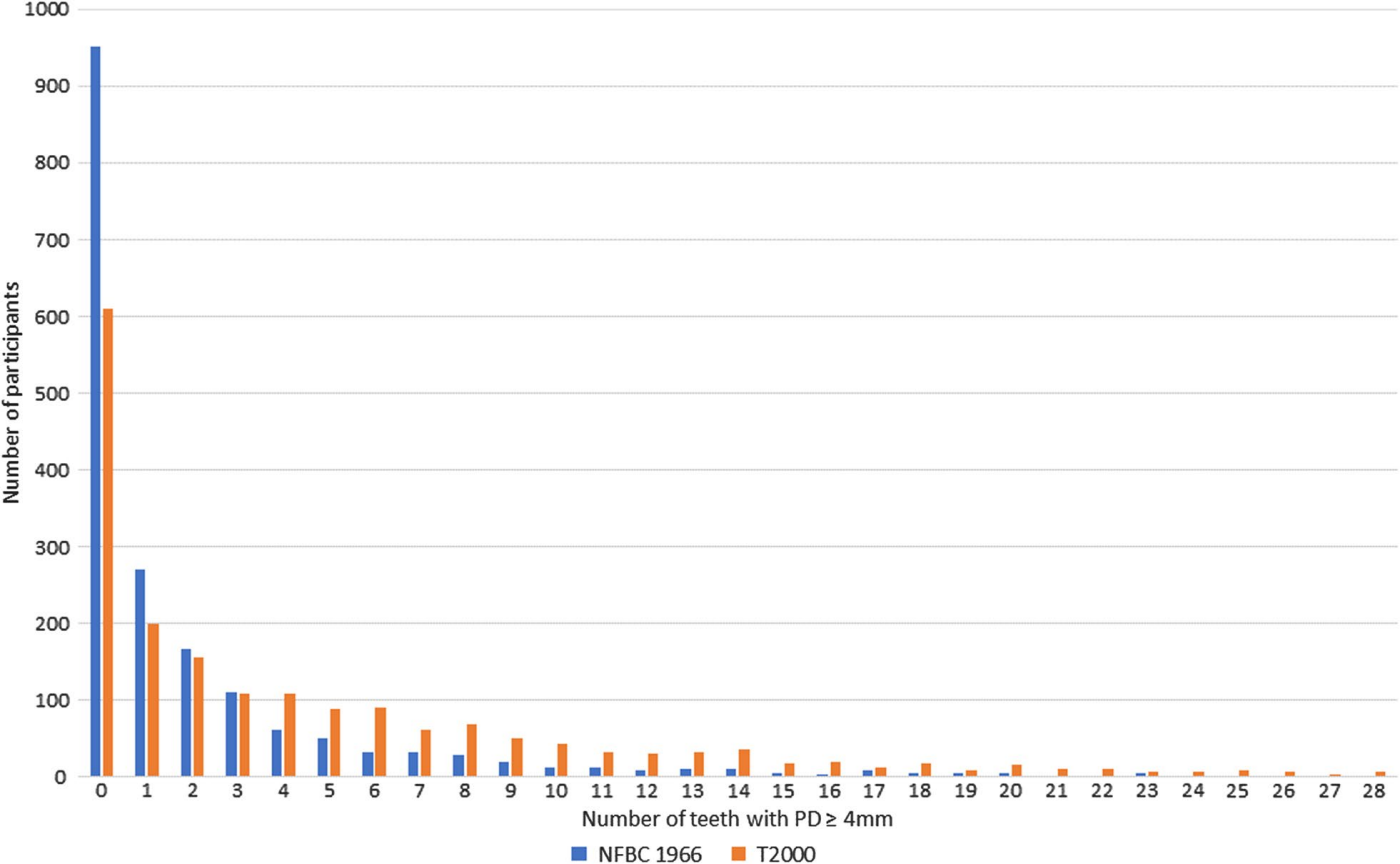
Frequency distributions of the number of teeth with PD ≥ 4 mm

In this study, the aim was to investigate genome-wide associations of the number of teeth with deepened periodontal pockets (PD ≥ 4 mm) in 30–65-year old Finnish adults (Fig. 1). We used the data of two studies, the national Health 2000 Survey and the Northern Finland Birth Cohort 1966 Study (NFBC1966), collected using the following methods:

### The Health 2000 survey

In the Health 2000 Survey, which consisted of a study of persons living in mainland Finland in 2000–2001, a clinical oral health examination was carried out on 6,335 individuals aged 30 years or over [23, 24].

In the clinical oral health examination, dental plaque was measured from three predetermined teeth, on one surface each: from the buccal surface of the most posterior tooth in the upper right quadrant, from the lingual surface of the most posterior tooth in the lower left quadrant, and the labial surface of the lower canine in the lower left quadrant [25]. The presence of plaque was categorized into three categories: no visible plaque (value 1), visible plaque on gingival margins only (value 2), or visible plaque also elsewhere (value 3). The mean of these values was used as an individual plaque score in the statistical analyses.

Probing pocket depths were measured using a ball-pointed WHO periodontal probe with markings at 3.5 and 5.5 mm. Measurements were taken at four sites per tooth (distal angle on the buccal side, midbuccal, midlingual and mesial angle on the lingual side). Only the deepest pocket on each tooth was recorded as follows: periodontal pocket 4–5 mm deep and ≥ 6 mm deep. A variable, the number of teeth with ≥ 4 mm deep periodontal pockets, was then created for statistical analyses. Third molars and radices were not included in the periodontal examination.

### The NFBC 1966 study

The NFBC 1966 Study is a life-span cohort study of individuals born in 1966 in the two northernmost provinces in Finland [26]. In 2012–2013, when the subjects were 46 years old, a comprehensive oral health examination was included in the study for the first time. It was carried out on 1,964 persons living in Oulu or within a 100 km radius from Oulu, the largest city in northern Finland.

In the periodontal examination, plaque was registered from the buccal sites of the teeth as follows: no visible plaque (value 0), plaque when lightly touching the tooth surface by the tip of a periodontal probe (LM 8-520B, Lääkintämuovi, Finland) or visible plaque (value 1). For the statistical analyses, the figures were converted into a percentual plaque score, i.e. percentage of sites with value 1, to better correspond to the scale used in the Health 2000 Survey (1–3, continuous variable). Probing pocket depths were measured at four sites per tooth (mesiobuccal, midbuccal, distobuccal and midoral). The probing force was calibrated for every subject using a letter scale (corresponding to 25 g). The number of teeth with ≥ 4 mm deep periodontal pockets was calculated for the statistical analyses. Wisdom teeth and radices were excluded from the periodontal examination.

Subjects with diabetes mellitus (T1DM or T2DM) and rheumatoid arthritis were excluded from both surveys based on a health interview of the Health 2000 Survey and an oral glucose tolerance test (screened T2DM) and questionnaire data on general health of the NFBC 1966 Study. In addition, we excluded subjects over the age of 65 from the Health 2000 Survey. In both surveys, data on smoking were collected using data of the health interview and the questionnaire and categorized as non-smoker or smoker (including both former and current smokers).

The basic characteristics of altogether 3,906 participants in the two surveys are presented in Table 1.

**Table 1:** Basic characteristics of the study populations

	Health 2000 Survey (N = 2068)	NFBC 1966 Study (N = 1838)
Age, years	30–65	46
Sex		
Female	51.5	53.5
Male	48.5	46.5
Smoking status		
Non-smoker	70.6	68.9
Current smoker	29.4	21.4
Missing data	0.0	9.7
Plaque^c^, mean (SD)	1.8 (0.7)^a^	1.4 (0.6)^b^
Number of teeth with PD ≥ 4 mm^d^, mean (SD)	4.7 (5.9)	2.0 (3.9)

SD standard deviation, PD pocket depth

^a^The mean of maximum plaque values (1, 2 or 3)

^b^Scale 1–3

^c^Data available of 1560 participants in the Health 2000 Survey and of 1809 participants in the NFBC 1966 Study

^d^Data available of 1860 participants in the Health 2000 Survey and of 1820 participants in the NFBC 1966 Study

### Genotyping

The Health 2000 DNA samples were genotyped using Illumina Humanhap 610 k array and the NFBC 1966 using Illumina Humanhap 310 k array. The genotypes were called with Illuminus software and imputed to 1000 g reference (phase 1 v3) using IMPUTE2 software (http://mathgen.stats.ox.ac.uk/impute/impute_v2.html). The following exclusion criteria were used in the quality control of original datasets: NFBC 1966: Call rate in the final sample was < 95%, P value of Hardy–Weinberg Equilibrium (HWE) < 0.0001, and MAF < 1%; Health 2000: Call rate < 95%, HWE < 1 × 10^–6^, MAF < 1%. The more detailed information of sample preparation, genotyping and quality control have been thoroughly described in the first original GWA studies of the Health 2000 Survey [27] and the NFBC1966 Study [28].

### Statistical analysis

The SNP-TEST v 2.4.1, which is a test for the analysis of SNP association in GWAS (https://mathgen.stats.ox.ac.uk/genetics_software/snptest/snptest_v2.4.1.html), was used. Variants were filtered prior to the meta-analysis by minor allele frequency (MAF; < 0.01), additive model information measure (< 0.7) and SE > 0.

Genome-wide association studies were performed separately for the two studies using additive regression models adjusted for population stratification, age, sex, smoking, and plaque. The first ten principal components were used in the analyses for population stratification. The number of teeth with deepened periodontal pockets (≥ 4 mm) was used as a continuous outcome variable.

**Fig. 2:**
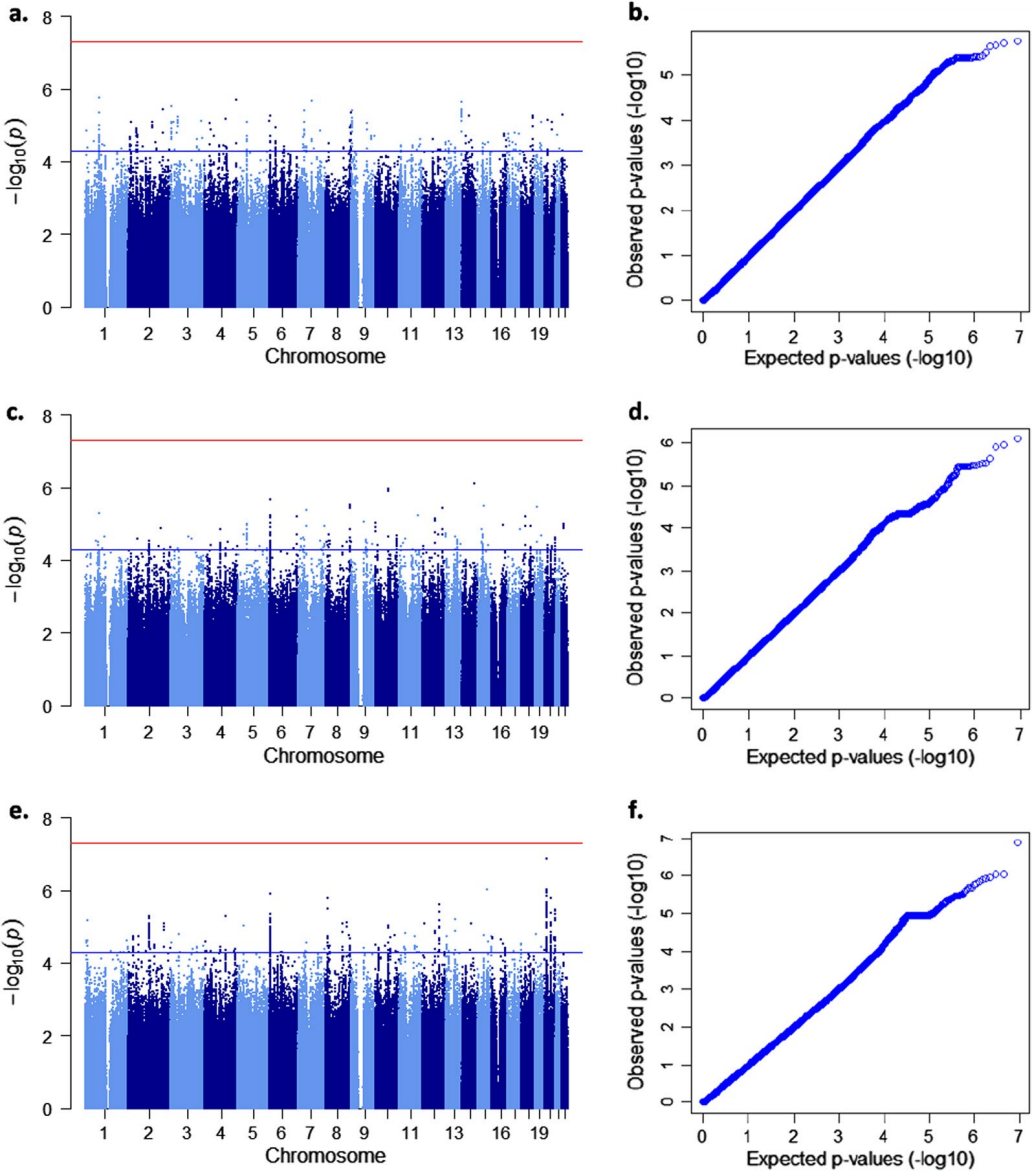
Manhattan and quantile–quantile plots showing GWAS results for the quantitative trait with three different adjustments. Manhattan (**a**, **c**, **e**) and quantile–quantile (**b**, **d**, **f**) plots related to the same adjustments are given side by side: **a**, **b** age, sex, and the first ten principal components. The genomic inflation factor lambda is 0.99 for NFBC66 and 1.01 for T2000 data. **c**, **d**: age, sex, smoking, and the first ten principal components. The genomic inflation factor lambda is 0.98 for NFBC66 and 0.99 for T2000 data. **e**, **f** age, sex, smoking, plaque, and the first ten principal components. The genomic inflation factor lambda is 0.98 for NFBC66 and 0.99 for T2000 data. Red lines indicate genome-wide signifcance level, p < 5 × 10^–8^

The results were combined in a meta-analysis applying a fixed effects model and using GWAMA, a software for performing genome-wide association meta-analysis (https://www.geenivaramu.ee/en/tools/gwama). Genomic control was enabled during meta-analysis. Manhattan plots (Fig. 2) were created using the qqman package for R software (https://cran.r-project.org/web/packages/qqman/index.html).

While a total of 3245 subjects were included in the final age- and sex-adjusted analyses the fully adjusted analyses were based on the data of 2650 subjects. The threshold for statistical significance on the genome-wide level was defined as *p* value < 5 × 10^−8^. Another threshold was set at p-level of 5 × 10^−6^ for reporting so called “suggestive” associations.

## Results

In the national Health 2000 Survey and the NFBC 1966 Study, 48.5% and 46.5% of the participants were males, and 70.6% and 68.9% non-smokers, respectively (Table 1). The mean number of teeth with ≥ 4 mm deep periodontal pockets was 4.7 (SD 5.9) in the Health 2000 Survey and 2.0 (SD 3.9) in the NFBC 1966 Study (Table 1).

While no significant genome-wide associations (*p* value < 5 × 10^–8^) with the number of teeth with ≥ 4 mm deep pockets were found in this study 17 loci, in total, reached the *p*-level of 5 × 10^–6^ in the analyses (Fig. 2, Table 2). We used three different sets of co-variates in the analyses and discovered that the SNP with the lowest *p* value, rs4444613 in chromosome 20 (*p* = 1.35 × 10^–7^), was found when a full set of co-variates including smoking and plaque was used (Table 2). We also observed that different top hits emerged when different co-variates were used. From beyond the top hits, only one SNP, rs200392355, emerged twice; in the fully adjusted analysis (1.22 × 10^–6^) and when adjusting for age, sex, smoking and the first ten principal components (2.23 × 10^–6^) (Table 2). There was not any observable sign of population stratification in the qq-plots. Qq-plots and respective lambda values are given in Fig. 2.

**Table 2:** Association results of the top SNPs in the meta-analysis

	*N*	Chr	Position	SNP	Nearest gene (function)	GTEX	Effect allele	EAF	Beta (95% CI)	SE	*P* value
(a)	3245	1	79069409	rs187209330	*IFI44L* (intergenic)		G	0.015	− 0.55 (− 0.78; − 0.32)	0.12	1.81 × 10^–6^
		2	201819623	rs867275005	*ORC2* (intron)		GAATA	0.040	− 0.33 (− 0.47; − 0.19)	0.071	3.83 × 10^–6^
		3	5859909	rs12494721	*AC027119.1*		T	0.14	0.18 (0.11; 0.26)	0.039	3.11 × 10^–6^
		4	183159364	rs12508476	*TENM3* (intron)		C	0.83	0.16 (0.096; 0.23)	0.034	2.01 × 10^–6^
		7	75505491	rs56045460	*RHBDD2* (upstream)	*TMEM120A* (skin)	A	0.088	− 0.23 (− 0.33; − 0.14)	0.049	2.17 × 10^–6^
		7	37556237	rs1364731	*ELMO1* (intergenic)	*NME8* (skin, thyroid)	T	0.37	0.12 (0.070; 0.17)	0.026	3.92 × 10^–6^
		9	6868737	rs77366980	*KDM4C* (intron)	*KDM4C,GLDC* (brain)	T	0.14	− 0.17 (− 0.25; − 0.10)	0.038	3.91 × 10^–6^
		13	112056064	rs7324141	*TEX29* (intergenic)	*ARHGEF7* (nerve, blood, adipose)	A	0.50	− 0.12 (− 0.17; − 0.069)	0.025	2.34 × 10^–6^
(b)	2925	6	7452509	rs200392355	*HNRNPLP1* (indel)		CT	0.46	0.14 (0.084; 0.20)	0.030	2.23 × 10^–6^
		8	139663617	rs3923549	*COL22A1* (intron)		G	0.91	− 0.21 (− 0.30; − 0.12)	0.045	3.01 × 10^–6^
		10	72481095	rs72814570	*ADAMTS14* (intron)	*ADAMTS14* (skin, mucosa, fibroblasts)	A	0.23	− 0.16 (− 0.22; − 0.093)	0.032	1.10 × 10^–6^
		14	91608044	rs147203970	*C14orf159* (intron)		T	0.033	0.37 (0.22; 0.52)	0.075	8.06 × 10^–7^
		15	59799643	rs16941452	*FAM81A* (intron)		G	0.022	− 0.45 (− 0.63; − 0.26)	0.096	3.24 × 10^–6^
(c)	2650	6	7452509	rs200392355	*HNRNPLP1* (indel)		CT	0.46	0.16 (0.093; 0.22)	0.032	1.22 × 10^–6^
		8	10958526	rs2409703	*XKR6* (intron)		C	0.079	0.28 (0.16; 0.39)	0.058	1.61 × 10^–6^
		15	76021782	rs11630851	*ODF3L1* (downstream)	*LMAN1L* (colon), *NRG4* (lung, pituitary)	T	0.067	0.30 (0.18;0.42)	0.061	9.39 × 10^–7^
		20	13340138	rs4444613	*BANF2* (intergenic)	*ISM1* (brain)	A	0.087	− 0.28 (− 0.38; − 0.18)	0.053	1.35 × 10^–7^
		20	37763743	rs2003705	*RP11-101E14.3* (intergenic)	*DHX35* (colon, oesophagus, testis), *FAM83D* (artery, colon, oesophagus, skin), *RP4-616B8.5* (oesophagus)	T	0.20	− 0.16 (− 0.23; − 0.097)	0.034	1.68 × 10^–6^

N, number of participants; Chr, chromosome; SNP, variant identifier; Beta, effect estimate (unit is the number of affected teeth); EAF, effect allele frequency; SE, standard error; GTEX, genotype-tissue expression (GTEX-portal); Adjustments (a) Age, sex, and the first ten principal components, (b) Age, sex, smoking status, and the first ten principal components, (c) Age, sex, smoking status, plaque, and the first ten principal components. Reference genome: GRCh37

## Discussion

In this study, which was the first GWAS on periodontal condition in the Finnish adult population, no statistically significant associations were found. However, in total 17 SNPs showed associations at the p-level < 5 × 10^−6^; of these rs4444613 was the stongest one (*p* = 1.35 × 10^−7^). In line with a number of earlier studies, which required statistical evidence of association at a *p* value level of < 5 × 10^−8^, we conclude that there were no associated genetic loci with the presence of deepened periodontal pockets (≥ 4 mm) in this population. It is likely that a larger sample size would be required to uncover genetic variation underlying the periodontal condition in this population.

In terms of earlier GWAS on chronic periodontitis, only a few loci have reached the genome-wide significance and hardly any of the findings have been replicated in an independent sample. Sanders et al. (2017) found an association with a rare variant of *TSNAXDISC1* noncoding mRNA (lead signal: rs149133391, MAF: 0.011) in a large population of Hispanics and Latinos and the variant was replicated in an African-American but not in a European-American sample. However, the finding should be interpreted with caution due to the rarity of the variant and small sample size in the replication data. In a genetically isolated population in Italy, four SNPs in the *EFCAB4B* gene (rs242016, rs242014, rs10491972, and rs242002) were found to be significantly associated with localized periodontitis but the findings have not been replicated so far [20].

Different variants of *SIGLEC5* were found as shared risk loci in studies combining AgP and severe CP cases in German, Dutch and Turkish populations [19], and rediscovered in a meta-analysis of German and Dutch AgP, and European-American and German CP cases [21]. In addition, significant variants for *DEF1A3* (rs2978951, rs2738058), *MTND1P5* (rs16870060), and *LOC107984137* near the *SHISA9* gene (rs729876) were found in these studies combining CP and AgP cases [19, 21]. From these genes, only *SIGLEC5* related variants have been found in later studies. Shungin et al. (2019) also found a single-risk locus of *SIGLEC5* (rs12461706, *p* = 3.9 × 10^−9^) by combining a questionnaire-based proxy phenotype of “loose teeth” (referring to severe periodontitis) with clinically verified periodontitis data. Also, the association of *SIGLEC5* related variants with severe periodontitis of rapid progression phenotype, stage III-IV, grade C according to the new periodontitis classification, was recently re-discovered in a pathway analysis but not in GWAS by de Coo et al. (2021).

Overall, the evidence for the genetic basis of CP based on the GWAS approach has so far been only modest. Moreover, the studies where SNPs exceeded the threshold of a significant association reported no SNPs in common, so there has been hardly any overlap in the genetic variants among the numerous suggestive associations reported to date [11, 13, 14]. In similar fashion, the 17 suggestive SNPs (*p* < 5 × 10^–6^) found in this study were not found in the GWAS catalog (https://www.ebi.ac.uk/gwas/), which lists in total l54 suggestive SNPs associated with periodontitis (published before 31th of August 2021). Neither were the previously reported significant *SIGLEC5* associated SNPs (rs4284742, rs11084095, rs12461706) linked with periodontal pocketing in our analysis (*p* > 0.3). It has also appeared that the significant gene variants linked with the susceptibility to periodontitis using the candidate gene approach [1] turned out to be non-significant in the GWAS approaches [8, 13, 14]. The main problem in the previous studies and also within our study is the limited sample size to uncover the genetic background of tens or hundreds of probable periodontal risk genes/ loci with low independent effect size and possible interactions with other risk factors.

Although the search for genome-wide significant variants has not produced consistent results, it does not mean that such variants do not exist. The stringent GWAS significance level minimizes the risk of false positives (type I error). However, in this study and other previous GWAS of periodontitis, it is evident that causative variants (with minor effect size) were classified statistically non-significant (type II error) because lack of statistical power. The omnigenic model/hypothesis of complex traits proposes that there is a huge number of common variants with a very small effect and, possibly, a small number of rare variants with moderate or larger effects. In GWA studies in periodontology, there are hardly any replicated variants with even moderate effect size (OR < 1.5) and it seems more likely that genetic risk of periodontitis is influenced by hundreds or thousands of genes with very small effect size [29, 30]. Apart from the variation in the DNA sequence, there may also be changes in gene expression. Growing evidence exists that epigenetic changes, which are not captured by GWASs, play a role in the inflammatory pathways of periodontitis through activation or inactivation of genes [31].

Teumer et al. (2013) reported that different top SNPs emerged for different case definitions of periodontitis in the same population [8]. The fact that various definitions for periodontitis were used partly explains the heterogeneity between the results of earlier genetic studies. Commonly used measures for periodontal tissue destruction include periodontal attachment level, probing pocket depth, the latter two combined, and radiographic bone loss. In this study, we used the number of teeth with periodontal probing depth ≥ 4 mm as a surrogate measure of existing inflammatory periodontal condition. A disadvantage of the outcome variable is that it does not take into account possible gingival retraction, which is common in older individuals, and therefore may underestimate the severity of periodontitis in the population. Due to lack of data on attachment level (Health 2000 Survey, NFBC1966 Study) and alveolar bone level (Health 2000 Survey) we could not identify true periodontitis cases and, instead used the number of teeth with deepened periodontal pockets (PD ≥ 4 mm) as a continuous outcome variable. The advantage of the continuous variable is that it comprises and utilizes information of the extent of periodontal disease. Additional advantage is that it reduces the possibility of misclassification of periodontal condition when compared with a situation where a dichotomous outcome is used.

The new periodontitis classification system, based on stages of clinical attachment loss and treatment complexity, and grades of disease progression rate [32] may help in standardizing periodontitis phenotypes. However, the age of the disease onset is not included in the new classification, which poses challenges to identify cases with high genetic risk, i.e. severe cases with rapid disease progression or patients with disproportionally rapid disease progression. Additional challenge is that the category to which those genetically susceptible periodontal patients often belong (Stage III-IV, Grade C) is the same to which the patients who are heavy smokers or patients with poorly controlled diabetes often belong.

To increase homogeneity of study population we excluded individuals with diabetes mellitus and rheumatoid arthritis due to their links with periodontitis [33, 34]. In addition, to reduce the effect of tooth loss in the older part of the population, the study population was restricted to subjects under 66 years of age in the Health 2000 Survey. In light of earlier studies, which have shown that, compared to older age groups, heritability has a stronger effect on periodontal condition in subjects aged < 65 years [7] or ≤ 60 years [8] the latter restriction is relevant.

Sex, age and smoking, but not dental plaque, are commonly controlled for in GWAS. In this study, we controlled also for the presence of dental plaque, which can be justified based on previous studies where dental plaque has consistently been associated with gingival inflammation, disease progression, treatment failure, and disease recurrence [35–37]. Visible dental plaque and the number of deepened periodontal pockets has also been shown to correlate within the Health 2000 data [38]. The above periodontal disease determinants were controlled for in the analyses by conducting three different modellings in a step-wise manner. It is worth noting that different variants were found depending on the set of used covariates (Table 2). It is assumed in GWAS that these disease determinants are equally distributed in the categories of explanatory variables (ie. SNPs) and are therefore not confounding factors. However, adjustments for these factors increase statistical power by reducing residual variance.

## Conclusion

This study was the first GWAS on periodontal condition in the Finnish adult population based on the meta-analysis of two Finnish cohorts. While the results of the meta-analysis showed no significant genome-wide associations with deepened periodontal pockets (≥ 4 mm), 17 SNPs were found that can be tested in future studies using larger sample sizes.

## Data Availability

NFBC 1966 data is available from the University of Oulu, Infrastructure for Population Studies. Permission to use the data can be applied for research purposes via electronic material request portal. In the use of data, we follow the EU general data protection regulation (679/2016) and Finnish Data Protection Act. The use of personal data is based on cohort participant’s written informed consent at his/her latest follow-up study, which may cause limitations to its use. Please, contact NFBC project center (NFBCprojectcenter@oulu.fi) and visit the cohort website (http://www.oulu.fi/nfbc) for more information. Health 2000 data that support the findings of this study are available from the Finnish Institute for Health and Welfare. However, restrictions apply to the availability of these data, which were used under license for the current study, and are therefore not publicly available. Nevertheless, data are available from the authors upon reasonable request and with permission from the NFBC and the Finnish Institute for Health and Welfare.

## Abbreviations

CP: Chronic periodontitis
AgP: Aggressive periodontitis
GWA: Genome-wide association
GWAS: Genome-wide association study
NFBC 1966: Northern Finland Birth Cohort 1966
SNP: Single nucleotide polymorphism
TSNAXDISC1: Translin-associated factor X Disrupted in schizophrenia 1
EFCAB4B: EF-hand calcium binding domain 4B
SIGLEC5: Sialic Acid Binding Ig Like Lectin 5
DEF1A3: Defensin alpha 1–3
MTND1P5: MT-ND1 pseudogene 5
SHISA9: Shisa family member 9
GLT6D1: Glycosyltransferase 6 domain containing 1
